# Emergency department patients with suspected infection at risk of intensive care unit transfer: a case-control Study

**DOI:** 10.1186/1757-7241-21-S2-A24

**Published:** 2013-09-09

**Authors:** Marie K Jessen Pedersen, Julie Mackenhauer, Anne Mette S W Hvass, Hans Kirkegaard

**Affiliations:** 1 Research Center for Emergency Medicine, Aarhus University Hospital, Denmark; 2 CONSIDER Sepsis Network, Denmark; 3 Emergency Department, Regional Hospital, Hjørring, Denmark; 4 Department of Infectious Disease, Aarhus University Hospital, Denmark; 5 Department of Anaesthesiology and Intensive Care, Aarhus University Hospital, Denmark

## Background

Sepsis is a time critical diagnosis and early treatment in the Emergency Department (ED) is essential. A challenge faced by emergency physicians is determining which patients with suspected infection will deteriorate and should be admitted to an intensive care unit (ICU). The aim of this study is to describe the population of ED patients with suspected infection. Further to compare patients who die or are transferred to an ICU within 2 days to those remaining at primary wards.

## Methods

We performed a retrospective case-control study. Inclusion criteria were: age>18y having a blood culture drawn upon admission to the ED at Aarhus University Hospital (MVA, KVA or Skadestuen) Jan 1st-Dec 31st 2011. Patients were grouped by in-hospital course within the first 2 days. Cases had a combined endpoint of death or ICU-transfer within 2 days. Controls remained at primary wards or in the ED. Matching was 1:3 by age and admission month. Laboratory results, antibiotics and clinical data were collected. Odds ratio (OR) and 95% confidence interval [CI] were calculated.

## Results

Of 1578 patients, 140 cases were matched to 401 controls. Total in-hospital mortality was 9%. Predictors of ICU-transfer or death within 2 days included lactate>2.5 mmol/L (OR 11.78 [6.93-20.4]), creatinine>170mmol/L (OR 4.28 [2.50-7.32]), respiratory rate>20min-1 (OR 3.71 [2.38-5.77]), altered mental status (OR 5.87 [3.69-9.34]) and having a suspected infection with unknown focus upon arrival (OR 2.13 [1.42-3.20]). Having more than one in-hospital ward transfer within 48 hours increased the risk of ICU-transfer or death (OR 2.09 [1.34-3.28]). Cases were more likely to fulfill the SIRS criterias compared to controls: Heart rate 105min-1[82;125] vs. 92min-1[80;105], respiratory rate 25min-1[17;32] vs.18min-1[15;24], WBC 12.9[9.3;19.9] vs. 10.8[7.8;14.5] while median temperature was normal both for cases 37.7°C[36.8;38.5] and controls 37.9°C[37.1;38.6].

## Conclusion

Simple clinical and paraclinical variables in the ED can predict outcome within two days. Having more than one in-hospital ward transfer seems to influence patient outcome negatively. Fever was not present for the majority of both cases and controls questioning the value of initial temperature as a predictor of severe outcome. Further analysis is needed developing a prediction rule of death or ICU-transfer within 2 days.

